# Studying the Effect of Agglomerates on the Mechanical Enhancement of Polymer Nanocomposites Using a Semiempirical Model

**DOI:** 10.3390/nano16080477

**Published:** 2026-04-17

**Authors:** Evagelia Kontou

**Affiliations:** Mechanics Department, School of Applied Mathematical and Physical Sciences, National Technical University of Athens, Iroon Polytechniou 9, Zografou Campus, 15780 Athens, Greece; ekontou@central.ntua.gr

**Keywords:** CNTs, CNFs, hybrid nanocomposites, agglomerates, modeling

## Abstract

In the present work, the elastic modulus of several types of polymer nanocomposites has been analyzed with a semiempirical model which takes into consideration agglomerate formation and their impact on the nanocomposites’ mechanical performance. The nanocomposites under investigation were either hybrids with a combination of graphene oxide (GO) with multi-walled carbon nanotubes (MWCNTs) or carbon nanofibers (CNFs) at various loadings, or monofillers with varying nanoparticle sizes, at a constant nanofiller loading. In addition, the effect of the type of polymeric matrix on the same nanofiller combinations has been examined. The basic assumption of two phases, namely a matrix with finely dispersed nanoparticles coexisting with agglomerates, was analyzed. The elastic stiffness of the first phase was calculated by the Mori–Tanaka model, and hereafter a semiempirical model was utilized for the estimation of the agglomerates’ stiffness. Within the context of this model, it was shown that the agglomerates’ volume fraction, combined with the nanoparticles’ density, namely the nanoparticles’ volume fraction in the agglomerates and consequently the inclusions’/agglomerates’ enhanced modulus, may cause a substantial improvement in the Young’s modulus, which cannot be explained by conventional mechanical models. These results apply to both nanocomposite types, hybrids at various nanofiller loadings and monofillers with varying particle sizes.

## 1. Introduction

Over the last few years, high-performance materials have been designed and produced by the addition of micro- or nanofillers into a polymeric matrix [1,2]. Nanofiller types, which are usually employed, include silicon dioxide (SiO_2_) as spherical nanoparticles, clays as nanolayers, and several types of carbonaceous nanomaterials. The latter include carbon nanotubes (CNTs), carbon nanofibers (CNFs) and graphene oxide (GO), all of which have attracted significant attention for a variety of applications due to their exceptional structures and remarkable properties. These nanofillers’ properties can be summarized as a high modulus and strength [3], a high aspect ratio [4], and significant electrical conductivity [5]. Furthermore, the simultaneous dispersion of two different nanofiller types into a polymeric matrix was shown to develop synergy between the matrix and the nanofillers, leading to interesting results [6,7,8,9,10].

The dispersion quality of nanofillers in a polymeric matrix was proved to have an impact on the ultimate characteristics of the nanocomposites, with fine nanofiller incorporation providing improved mechanical, thermal and electrical properties. It was found that at low nanofiller loadings, a homogeneous dispersion can be achieved, resulting in impressive enhancement [11,12,13].

However, with increasing nanofiller loading, and due to the inherent tendency of hydrophobic nanofillers to form clusters, the homogeneous fine dispersion is disrupted. This consequence may have a detrimental effect on the nanocomposites’ performance, mainly because the adhesion between clusters and polymer is disrupted. Additionally, as mentioned in [1,14], nanofiller clusters are described by the terms agglomerates or aggregates. More specifically, the term agglomeration denotes a cluster of nanoparticles that are tightly connected, whereas aggregation refers to a cluster of nanofillers that are feebly linked to each other. Therefore, it can be assumed that an aggregation is a reversible nanofiller assemblage [15], while the assembly of nanofillers described as agglomeration is not reversible [14]. In another work [16], the distinction between agglomeration and aggregation is based on the size of clusters in the nanocomposites.

The reinforcing mechanism of polymer particulate composites has been the subject of numerous works by means of micromechanical models evaluating the elastic constants of composites with varying volume fractions [17,18,19,20,21]. Particularly, modeling of the elastic properties of CNT/polymer nanocomposites has been extensively studied [22,23]. The Mori–Tanaka method was employed by Odegard [23] to calculate the elastic properties of CNT/polyimide composites at various lengths, orientations, and volume fractions. The mechanical improvement of polymer/CNT nanocomposites by analyzing the Young’s modulus and the yield strength of the matrix–filler interfacial region was studied in [24]. Within this context, the modeling of the interface/interphase region between the CNT and the polymer is reviewed in [25]. The theoretical studies are divided into two main groups: atomistic modeling and continuum modeling. In [26], the Young’s modulus of graphene/polymer nanocomposites was evaluated in terms of a full-range multi-scale approach covering the nano-, micro-, meso- and macroscales in the framework of a bottom-up hierarchical technique. In another approach [27], a model was developed based on a modified three-phase Mori–Tanaka model, considering fillers, agglomerates and matrix areas. A Monte Carlo method to disperse the fillers and detect agglomerations was employed. Hereafter the Reuss model was used to calculate the modulus of the agglomerates. It was then possible to predict the Young’s modulus of the composite. A satisfactory agreement between the model and experimental results for the elastic modulus of the materials under examination was found. The proposed model could be implemented with various nanocomposites, requiring, however, parameters such as orientation and distribution of the fillers. Therefore, transmission electron microscopy or machine learning should be employed.

Regarding the effect of agglomerates/aggregates on the mechanical enhancement of the polymer nanocomposites, a very limited number of studies have been published so far. In [28], the importance of exploring the dispersion and aggregation of nanoparticles by certain experimental techniques is highlighted. Coarse-grained molecular dynamics was adopted to analyze the procedure of aggregation of nanoparticles into polymer melts.

Within the same context, the impact of the agglomerates/aggregates on the mechanical enhancement of the polymer nanocomposites has been studied in [29]. The content and size of the agglomerates/aggregates could be defined by employing a method analyzing the equilibrium between the dispersion and cohesion energies in the preparation stage. The effect of agglomerates on the physical properties of polymers/nanocomposites has been investigated in [30,31].

Several reasons and related mechanisms may lead to the formation of agglomerates/aggregates in a system. In the case of carbonaceous nanocomposites based on CNTs or graphene, the formation of agglomerates mainly takes place during the fabrication procedure [2]. On the other hand, in the case of composites comprising micro-size particles, it has been specified that the agglomerations regenerate when the repulsive forces between them are sufficiently reduced [32].

It has been reported [33] that despite several works having been published on polymer nanocomposites with well-dispersed graphene nanofillers, only a few analyze the agglomerates’ formation and their impact on the thermomechanical performance of the materials. The prediction of the elastic modulus of nanocomposites, including well-dispersed, partly agglomerated and fully agglomerated graphene nanoparticles, was achieved by a multi-scale model [34]. The results of this novel multi-scale modeling approach were more accurate compared with previously developed stochastic modeling. Within the same context, the effect of agglomerates has been studied either experimentally or by mathematical models and numerical analysis in [35,36]. In [36] different mathematical models are employed to describe the Young’s modulus of epoxy/nanocomposites. It was shown that the interphase and agglomerates are crucial to understanding the nanocomposites’ reinforcing mechanism. The elastic modulus of polymer hybrid/carbonaceous nanocomposites has been studied by finite element analysis (FEA), considering agglomerate formation with CNT curvature [37]. In addition, FEA has been employed to study the thermomechanical properties of polymer nanocomposites [38].

In the present work, a semiempirical two-phase model was utilized to describe the effect of agglomerate formation on the elastic modulus of a variety of polymer/nanocomposites. The first phase is a matrix with a portion of fully dispersed nanoparticles, and the second one is the agglomerated nanoparticles, the so-called inclusions. The development of agglomerates is a very common effect in nanocomposites, as presented above, and is usually encountered as a reason for degradation. A rather limited number of works have dealt with this effect. The inclusions’ volume fraction takes the role of an effective volume fraction with a polymeric matrix and nanoparticles.

In our analysis, the employed model reveals the effect of the nanofillers’ loading, the matrix and nanofiller type, and the inclusions’ volume fraction and their density in nanoparticles on the mechanical enhancement achieved. The model’s assumptions indicate an enhanced modulus of the inclusions, which explains the reinforcing mechanism of the agglomerates. The model results for various types of hybrid nanocomposites and for monofillers with varying nanoparticle sizes converge to show the decisive role of these factors. Notwithstanding the existence of sophisticated models for agglomerates, the present analysis, due to its simplicity and the confirmed convergence of various experimental results, provides encouraging findings, and could be the basis for further research.

### Model Presentation

**Figure 1:**
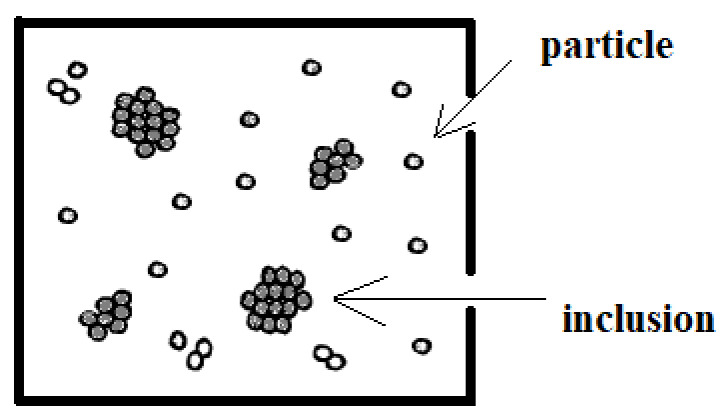
Schematic presentation of the inclusion model with agglomerated and fully dispersed nanofillers.

The semiempirical analytical model adopted in the present work to describe the elastic modulus of a polymer nanocomposite material entails the presence of two phases [39,40]. The polymer nanocomposite consists of a polymeric matrix with both fully dispersed nanoparticles and agglomerates of nanoparticles which may also include areas of polymeric bulk, as illustrated in Figure 1.

The first phase is called the fictitious matrix, and the agglomerates are given the general term inclusions. It is also assumed that the inclusions are of a spherical shape, regardless of the specific geometry of the involved nanofillers. In our analysis, the elastic constants of the fictitious matrix (matrix + nanofillers) will be evaluated by the analytical model by Mori–Tanaka and Benveniste [18,21].

The fictitious matrix stiffness tensor **C** is given by

(1) $$C=C_{m}+V_{f}\left[(C_{f}-C_{m})\;A_{f}\right]+{\left[V_{m}I+V_{f}A_{f}\right]}^{-1}$$

where **C_m_** is the polymer’s stiffness tensor, **C_f_** the nanofillers’ stiffness tensor, V_f_ the nanofiller’s volume fraction, V_m_ the polymeric matrix volume fraction, **I** the identity tensor, and **A_f_** the so-called stress concentration tensor, given by

(2) $$\begin{array}{l} A_{f}={\left[I+S\;(C_{m}{)}^{-1}\left(C_{f}-C_{m}\right)\right]}^{-1} \end{array}$$

where **S** is the Eshelby tensor [20]:

(3) $$S=\left[\begin{array}{llllll} s_{11} & s_{12} & s_{13} & 0 & 0 & 0\\ s_{21} & s_{22} & s_{23} & 0 & 0 & 0\\ s_{31} & s_{32} & s_{33} & 0 & 0 & 0\\ 0 & 0 & 0 & s_{44} & 0 & 0\\ 0 & 0 & 0 & 0 & s_{55} & 0\\ 0 & 0 & 0 & 0 & 0 & s_{66} \end{array}\right]$$

which for spherical inclusions is expressed by

(4) $$\begin{array}{l} s_{11}=s_{22}=s_{33}=\frac{7-5\nu_{0}}{15\left(1-\nu_{0}\right)} \end{array}$$

(5) $$s_{12}=s_{23}=s_{31}=-\frac{1-5\nu_{0}}{15(1-\nu_{0})}$$

(6) $$s_{44}=s_{55}=s_{66}=\frac{4-5\nu_{0}}{15(1-\nu_{0})}$$

where ν_0_ is the Poisson’s ratio of the polymeric matrix.

The assumption of spherical inclusion seems to be a rough approximation, but it is close to reality, as can be seen in SEM pictures presented in previous works [9,10,41].

The elastic modulus of the inclusions will be calculated by the Tsai–Pagano micromechanics model, assuming good adhesion between matrix and nanofillers, good dispersion quality and random distribution of the nanofillers. The modulus of elasticity of the inclusions E_incl_ is given by the modified rule of mixtures:

(7) $$E_{\mathrm{incl}}=E_{f}\lambda V_{f}+E_{m}{(V}_{\mathrm{incl}}-\lambda V_{f})$$

where E_f_, E_m_ are the Young’s moduli of the nanofillers and the polymeric matrix correspondingly. The quantities λ and V_incl_ are model parameters defined as

(8) $$\lambda =\frac{V_{f}^{\mathrm{incl}}}{V_{f}}$$

where $V_{f}^{\mathrm{incl}}$ is the nanofillers’ volume fraction in the inclusion; therefore, parameter λ denotes the fraction of nanofillers contained in the inclusions per the total nanofiller volume fraction. Parameter V_incl_ is the inclusion volume fraction. Equation (7) actually expresses the rule of mixtures for the iso-strain condition. For the model to be valid, parameters λ and V_incl_ should satisfy the following expressions:

(9) $$V_{f}^{\mathrm{incl}}<V_{\mathrm{incl}}\;\;\;\;\;\;\;\;\;\;\;\;\;\;\mathrm{and}\;\lambda <\frac{V_{\mathrm{incl}}}{V_{f}}$$

In addition, the following inequalities should be valid: 0 < λ and V_incl_ < 1.

The elastic modulus of the nanocomposite E_c_ will be finally given by

(10) $$E_{c}=E_{\mathrm{incl}}V_{\mathrm{incl}}{+(1-V}_{\mathrm{incl}}{)\;E}_{m}^{f}$$

where $E_{m}^{f}$ is the Young’s modulus of the fictitious matrix, calculated by Equation (1).

It will be shown in the following that the parameters V_incl_ and λ or $V_{f}^{\mathrm{incl}}$ play a crucial role in understanding the reinforcing mechanism in the nanocomposites.

**Figure 2:**
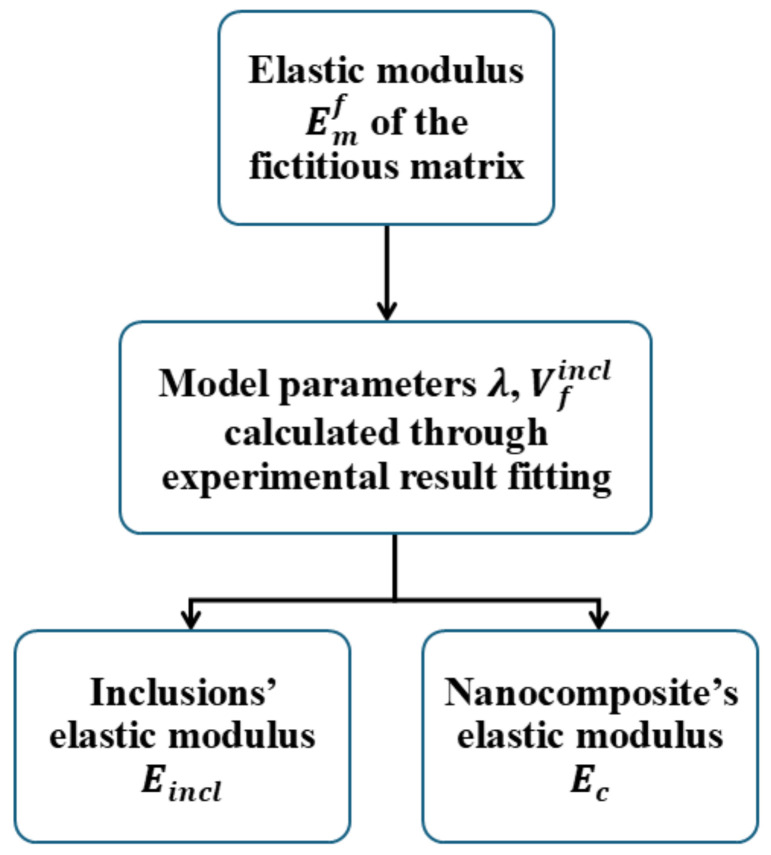
Flow diagram of model calculations.

The model calculation procedure is schematically shown in the flow diagram in Figure 2.

## 2. Materials and Experiments

The first series of polymer/nanocomposites analyzed with the present model consists of a linear low-density polyethylene matrix produced by a metallocene catalyst, designated as mLLDPE, reinforced with a blend of graphene oxide (GO) and CNTs or CNFs at an equal ratio and three total weight loadings, namely 1.31, 3.84 and 6.25 wt.%, as extensively studied in [9]. Their designation and the Young’s modulus experimental results are shown in Table 1.

The second series of nanocomposites examined is based on polylactic acid (PLA) reinforced with a blend of graphene oxide (GO) and CNTs or CNFs at an equal ratio and three total weight loadings, namely 3.84, 6.25 and 8.0 wt.%, as extensively studied in [10]. The PLA/hybrid designation and the elastic modulus experimental results are shown in Table 1. Model simulation results for both series are shown in Table 2.

The third series of polymer nanocomposites includes polymers reinforced with SiO_2_ nanoparticles of varying size, at the same weight fraction, 4 wt.%. Two polymers were employed, namely PLA and an epoxy resin under the commercial name ES-35. The sizes of the SiO_2_ particles ranged from 13 to 22, 15 to 35, 18 to 35, and 55 to 75 nm and 0.5, 1.0 and 1.5 μm. The nanocomposites’ designation and their tensile modulus are presented in Table 3, while the preparation details and the experimental study are presented in [41].

## 3. Model Validation

### 3.1. Polymer Hybrid/Nanocomposites

The first class of hybrid nanocomposites analyzed with the present model consists of materials based on mLLDPE reinforced with a blend of either GO/CNTs or GO/CNFs at equal weight fractions, and at three total nanofillers’ loadings. These hybrid nanocomposites were extensively investigated in [9]. The Young’s modulus experimental results are shown in Table 1. The density of the mLLDPE was 0.9 g/cm^3^ and the density of the carbonaceous nanofillers was taken as equal to 1.43 g/cm^3^. Referring to these data and following Equations (1)–(7) and (10), according to the flow diagram in Figure 2, model parameters V_incl_ and λ were evaluated. It should be noted that a spherical shape was assumed for the inclusions, while the nanofillers’ Young’s modulus was taken to be equal to 100 GPa. In Table 2, the model parameter values and the simulated Young’s modulus of the nanocomposites are presented. It can be observed that a very close approximation between the experimental and model-calculated Young’s modulus has been achieved.

**Figure 3:**
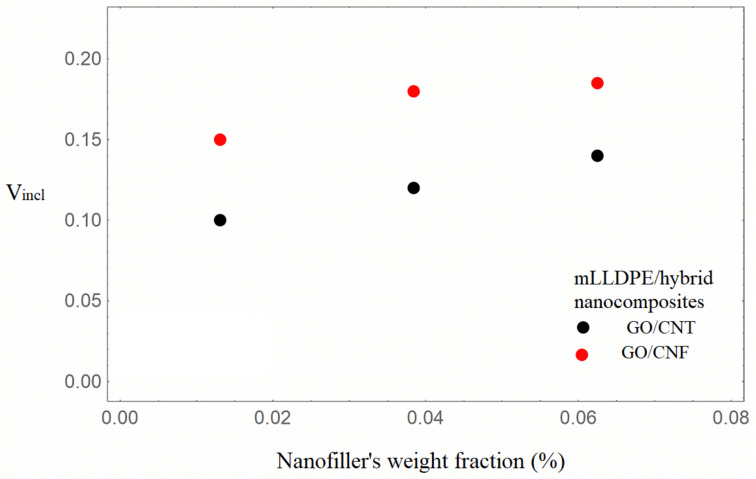
Inclusion volume fraction with an increasing total nanofiller weight fraction of the mLLDPE/carbonaceous hybrid nanocomposites.

**Figure 4:**
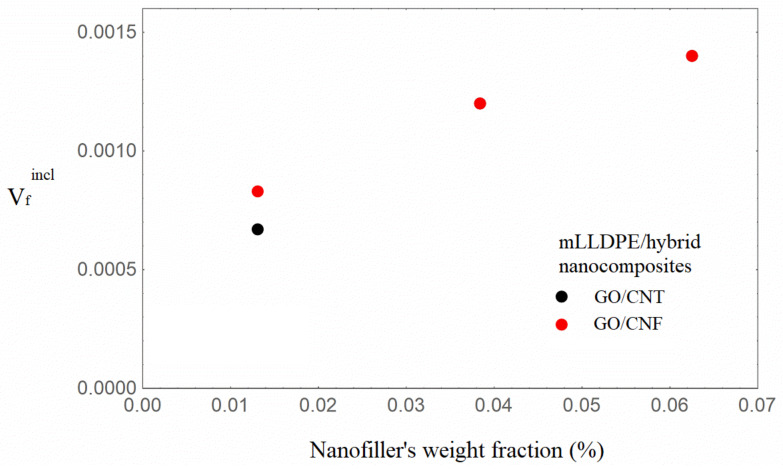
Nanofiller volume fraction in the inclusion with a varying total nanofiller weight fraction of the mLLDPE/carbonaceous hybrid nanocomposites.

It can be observed from Table 1 that the Young’s modulus increases with increasing nanofiller loading, with the hybrids based on GO/CNFs exhibiting the highest enhancement at similar nanofiller loadings. It can also be seen from Table 2 that the model parameter V_incl_, which expresses the inclusion volume fraction, increases with increasing nanofiller loading for both hybrid nanocomposite types. The situation of uniform dispersion of the nanofillers into the bulk matrix and in the inclusions, which is associated [40] with the condition V_incl_ = λ, is approximated for the content of 1.31 wt.%. In Figure 3 and Figure 4, the inclusions’ volume fraction and the nanofillers’ volume fraction in the inclusions versus the total nanofiller weight fraction are illustrated.

It can be observed from Figure 3 and Figure 4 that the inclusion volume fraction as well as the nanofillers’ volume fraction in the inclusions increase with an increase in the total weight fraction of nanofillers. The inclusion volume fraction is higher in the GO/CNF hybrids, whereas the nanofiller volume fraction in the inclusions is almost the same. Given that the mLLDPE/GO/CNF nanocomposites exhibit the greatest enhancement, it can thus be determined that the presence of inclusions (agglomerates/aggregates) does not necessarily have a detrimental effect on the nanocomposites’ reinforcement. The increment in the inclusions’ Young’s modulus E_incl_ with increasing total nanofiller content, denoting regions rich in nanofillers, seems to be a possible mechanism of total mechanical enhancement. The nanofillers’ partial final dispersion coexisting with inclusions rich in nanofillers may be considered as the two mechanisms leading to an augmented modulus of elasticity. It is worth noting that for partially or fully agglomerated graphene polymer nanocomposites, deterministic modeling [34] resulted in augmented values of the Young’s modulus, higher than the experimental ones.

The second series of polymer nanocomposites examined are based on polylactic acid (PLA) reinforced with a blend of GO/CNTs or GO/CNFs at equal weight ratios and at three different total nanofiller loadings. The PLA density is 1.24 g/cm^3^ and the nanofiller density was taken as equal to 1.58 g/cm^3^. These materials were prepared and experimentally studied in [10]. The Young’s modulus experimental results for the PLA/hybrid nanocomposites, namely PLA/GO/CNT and PLA/GO/CNF, are presented in Table 1.

**Figure 5:**
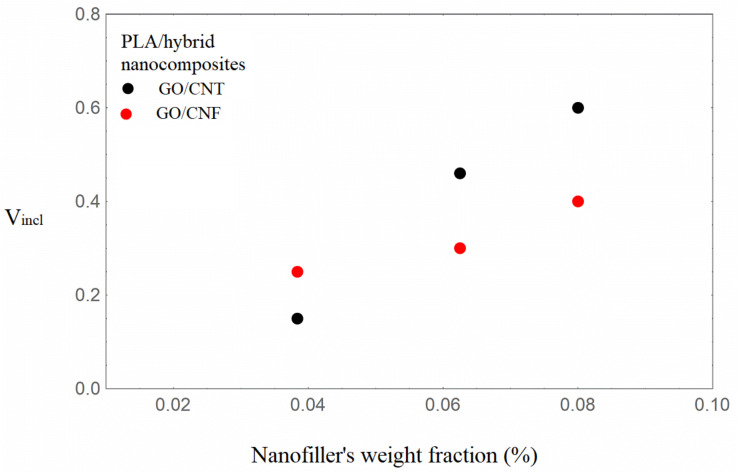
Inclusion volume fraction with an increasing total nanofiller weight fraction of the PLA/carbonaceous hybrid nanocomposites.

**Figure 6:**
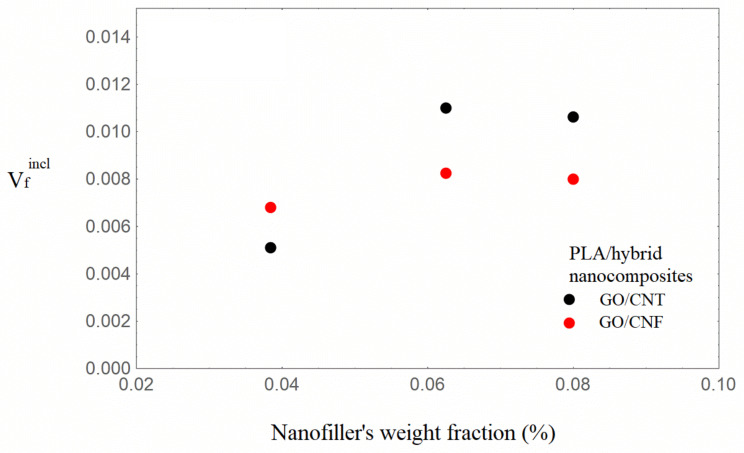
Nanofiller volume fraction in the inclusion with an increasing total nanofiller weight fraction of the PLA/carbonaceous hybrid nanocomposites.

The highest Young’s modulus increment is obtained for the PLA/GO/CNT hybrids at the two highest nanofiller loadings. The model-calculated elastic modulus values are very close to the experimental ones, as shown in Table 2. In Figure 5 and Figure 6, the inclusion volume fraction V_incl_ and the $V_{f}^{\mathrm{incl}}$ versus the total nanofiller weight fraction are comparatively shown for the two types of hybrids. Again, the general trend is that the inclusion volume fraction and the $V_{f}^{\mathrm{inc}}$ increase with increased total nanofiller loading.

Following the experimental results for the two series of hybrid nanocomposites, based on two different polymeric matrix types, it is revealed that the percentage of elastic modulus increment in the mLLDPE/nanocomposites is higher for the mLLDPE/GO/CNF hybrids, while in the PLA/hybrids it is higher for the PLA/GO/CNT nanocomposites. When employing the semiempirical inclusion model for the two nanocomposite series, in both cases the inclusion volume fraction is higher for the highest mechanical augmentation, with that for mLLDPE/GO/CNF/6.25 being equal to 0.185 and that for PLA/GO/CNT/8.0 being equal to 0.6.

Furthermore, the incremental trend of the inclusion volume fraction and the nanofiller volume fraction in the inclusions with increasing total nanofiller loading is verified for all polymer/hybrid nanocomposites investigated. The interplay between these parameters leads to a high E_incl_ value, which contributes to the total Young’s modulus enhancement.

### 3.2. Polymer/Silica Nanocomposites—The Effect of Particle Size

In this section, the effect of nanoparticle size on the mechanical enhancement will be analyzed within the frame of the model under consideration. Two series of nanocomposites based on PLA and an epoxy resin, reinforced with silica nanoparticles of varying size, at the same weight fraction of 4 wt.%, will be investigated. These materials have been extensively studied in [41]. The average silica diameter size ranges between 13 and 22, 15 and 35, 18 and 35, and 55 and 75 nm. In addition, three diameters at the micron scale, namely 0.5, 1.0 and 1.5 μm, were investigated. The density of the SiO_2_ particles was 2.0 g/cm^3^ and the volume fraction was 0.025. The materials’ designation and the elastic modulus experimental results for the silica/nanocomposites are presented in Table 3. In the same table, the simulated model parameters are also presented.

It can be seen from Table 3 that the deviation between the experimental and model-simulated Young’s modulus values is very low.

In general, for a perfect nanoparticle dispersion, a large increment in the Young’s modulus with decreasing particle diameter is expected. As shown in Table 3, in the PLA/silica nanocomposites no monotonic dependence of the elastic modulus enhancement on the particle size is observed, and this effect is strongly dependent on the agglomerates’ formation. The inclusion volume fraction is rather constant and equal to 0.7 at various particle sizes at the nanoscale and is constant with a different value at the micron scale. Regarding $V_{f}^{\mathrm{incl}}$ a non systematic trend can be observed.

The 20% modulus enhancement of PLA/15-35 is achieved with the constant value V_incl_ = 0.7, but a higher inclusion density in the nanoparticles, i.e., parameter λ = 0.7, is required. The 27% modulus increment for PLA/18-35 is described at the higher V_incl_ equal to 0.9, which denotes a more homogeneous dispersion [40], with the constant value of parameter λ = 0.5. It can be determined that the mechanical enhancement is indicated by the presence of inclusions counter-balanced with an appropriate high value of their density in nanoparticles, namely $V_{f}^{\mathrm{incl}}$^.^

In the micrometer-scale PLA/silica materials, the low to moderate modulus enhancement can also be described in a similar way.

**Figure 7:**
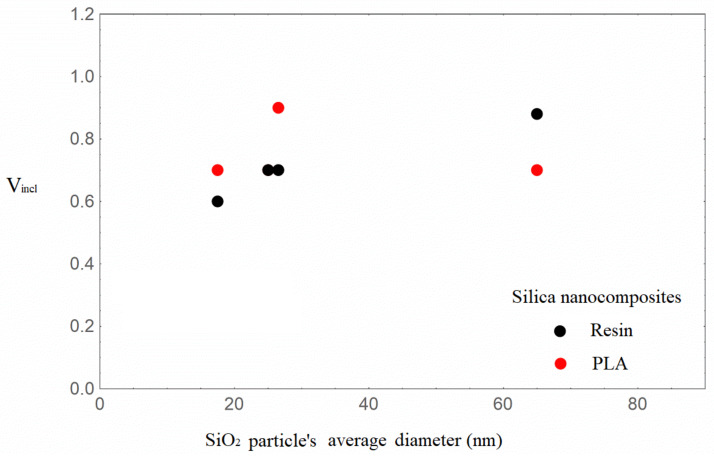
Inclusion volume fraction with an increasing average diameter of silica nanoparticles in the PLA and resin/SiO_2_ nanocomposites.

The resin/silica nanocomposites, which exhibit higher Young’s modulus augmentation compared to the PLA/silica ones, confirm the interplay between the model parameters V_incl_ and λ. The incremental trend of V_incl_ with increasing nanoparticle size, as shown in Figure 7, is accompanied by a rather monotonic elastic modulus increment (Table 3). At the highest elastic modulus enhancement, i.e., 60% for Res/55-75, it can be observed that high values of V_incl_ and parameter λ are required. It could thus be summarized that the inclusions’ volume fraction increment denotes a rather uniform particle dispersion, while the nanofillers’ density in the inclusions is also a crucial factor, and both effects contribute to mechanical enhancement.

In the resin/silica composites at the micrometer scale, no substantial differences are obtained regarding the elastic modulus enhancement or, consequently, the model parameter values.

## 4. Conclusions

In the present research, a modified semiempirical model is employed to describe the elastic modulus of polymer nanocomposites. The main concept of the model is the presence of two phases, so-called inclusions, which contain agglomerated nanofillers and matrix, and the presence of the fictitious matrix, i.e., a bulk polymer with mostly finely dispersed nanofillers. A well-known micromechanics model is utilized to calculate the elastic stiffness of the fictitious matrix, while a modified rule of mixtures is used to calculate the elastic stiffness of the inclusions. The volume fraction of the inclusions as well as their density in the nanofillers are two crucial model parameters which give insight into the reinforcing mechanism of the nanocomposites. The interplay between these parameters leads to a high E_incl_ value which contributes to the total Young’s modulus enhancement. It is thus revealed that the inclusions may have a reinforcing and not a detrimental effect on the nanocomposites’ mechanical performance. Various types of hybrid/carbonaceous nanocomposites with different polymeric matrices as well as nanocomposites with different nanofiller sizes were investigated within the frame of this model. In all cases it was confirmed that the inclusions’ volume fraction and the nanofillers’ volume fraction in the inclusions increase with increasing total nanofiller loading for all the polymer/hybrid nanocomposites investigated.

More specifically, in the LLDPE/hybrid nanocomposites, the highest enhancement of the Young’s modulus was exhibited for the mLLDPE/GO/CNF/3.84% and mLLDPE/GO/CNF/6.25% nanocomposites. Within the context of the model, the combination of a high value of the inclusion volume fraction, namely 0.18 and 0.185, as well as a high density of nanofillers in the inclusions, expressed by parameter λ, was required. A similar trend was confirmed for PLA/GO/CNT/6.25% and PLA/GO/CNT/8%, with the highest mechanical enhancement. High values of V_incl_ equal to 0.46 and 0.6 correspondingly in combination with parameter λ values equal to 0.2 and 0.15 were fitted to describe the experimental Young’s modulus. It should be noted that the absolute values of parameters depend on the selected value of the carbonaceous nanofillers’ Young’s modulus. The importance of the contribution of the model parameters, however, remains the same, while the trend in the comparative investigation is retained.

Within the same context, interesting results were obtained for nanocomposites with constant nanofiller loading and varying nanofiller sizes in two polymeric matrices, namely PLA and an epoxy resin. With increases in the nanofillers’ average size, the elastic modulus exhibits a rather incremental trend for both series, with the exception of PLA/55-75. The inclusions’ volume fraction attains high values ranging from 0.7 up to 0.88 for the silica diameters at the nanoscale, requiring increased values of parameter λ or high values of the nanofillers’ volume fraction in the inclusions. Regarding the composites with silica diameters at the micrometer scale, no substantial differences in the mechanical enhancement and model parameters were found.

The semiempirical model, employed for a variety of polymer nanocomposites, appears to be a useful tool for the interpretation of the nanocomposites’ mechanical enhancement in view of the presence of agglomerates.

## Figures and Tables

**Table 1:** Material designation and tensile properties of the LLDPE [9] and PLA [10] hybrid carbonaceous nanocomposites examined.

Material	Total Nanofiller Weight Fraction	Total Nanofiller Volume Fraction V_f_	Young’s Modulus Experimental Results (MPa)	Modulus Increment (%)
mLLDPE	-	-	92 ± 2.6	-
mLLDPE/GO/CNT/1.31%	0.0131	0.0083	118 ± 4.1	28.3
mLLDPE/GO/CNT/3.84%	0.0384	0.0245	154 ± 5.8	67.4
mLLDPE/GO/CNT/6.25%	0.0625	0.04	180 ± 6.4	95.6
mLLDPE/GO/CNF/1.31%	0.0131	0.0083	142 ± 5.2	54.3
mLLDPE/GO/CNF/3.84%	0.0384	0.0245	184 ± 6.9	100.0
mLLDPE/GO/CNF/6.25%	0.0625	0.04	204 ± 7.9	121.7
PLA	-	-	3064 ± 150	-
PLA/GO/CNT/3.84%	0.0384	0.034	3218 ± 160	5.0
PLA/GO/CNT/6.25%	0.0625	0.055	4265 ± 230	39.2
PLA/GO/CNT/8.0%	0.08	0.07	5652 ± 300	84.5
PLA/GO/CNF/3.84%	0.0384	0.034	3460 ± 190	12.9
PLA/GO/CNF/6.25%	0.0625	0.055	3796 ± 190	23.9
PLA/GO/CNF/8.0%	0.08	0.07	4000 ± 212	30.5

**Table 2:** Model parameter values of the LLDPE and PLA/hybrid carbonaceous nanocomposites examined.

Material	Inclusion Volume Fraction V_incl_	Parameter λ	$V_{f}^{\mathrm{incl}}$	Inclusions’ Young’s Modulus E_incl_ (MPa)	Theoretical Young’s Modulus (MPa)
m LLDPE	-	-	-	-	-
mLLDPE/GO/CNT/1.31%	0.1	0.08	0.00067	341	119
mLLDPE/GO/CNT/3.84%	0.12	0.05	0.0012	623	160
mLLDPE/GO/CNT/6.25%	0.14	0.035	0.0014	713	186
mLLDPE/GO/CNF/1.31%	0.15	0.1	0.00083	429	145
mLLDPE/GO/CNF/3.84%	0.18	0.05	0.0012	628	193
mLLDPE/GO/CNF/6.25%	0.185	0.035	0.0014	717	214
PLA	-	-	-	-	-
PLA/GO/CNT/3.84	0.15	0.15	0.0051	2994	3262
PLA/GO/CNT/6.25	0.46	0.2	0.011	8446	4279
PLA/GO/CNT/8.0	0.6	0.15	0.01	7116	5668
PLA/GO/CNF/3.84	0.25	0.2	0.0068	6145	3490
PLA/GO/CNF/6.25	0.3	0.15	0.0082	5019	3885
PLA/GO/CNF/8.0	0.4	0.1	0.008	4744	3995

**Table 3:** Material designations, experimental tensile properties [41] and calculated model parameters of the PLA and resin/nanocomposites examined.

Material	Young’s Modulus Experimental Results (MPa)	Modulus Increment (%)	Inclusion Volume Fraction V_incl_	Parameter λ	$V_{f}^{\mathrm{incl}}$	Inclusions’ Young’s Modulus E_incl_ (MPa)	Theoretical Young’s Modulus (MPa)
PLA	3000 ± 120	-	-	-	-	-	-
PLA/13-22	3200 ± 140	7	0.7	0.5	0.0125	3312	3262
PLA/15-35	3600 ± 144	20	0.7	0.7	0.0175	3798	3602
PLA/18-35	3800 ± 185	27	0.9	0.5	0.0125	3912	3835
PLA/55-75	3202 ± 135	7	0.7	0.5	0.0125	3312	3262
PLA/0.5	3455 ± 131	15	0.6	0.8	0.02	3740	3502
PLA/1.0	3250 ± 130	8	0.7	0.4	0.01	3070	3092
PLA/1.5	3120 ± 123	4	0.6	0.6	0.015	3255	3210
Resin ES-35	2150 ± 107	-	-	-	-	-	-
Res/13-22	2800 ± 133	30	0.6	0.8	0.02	3247	2850
Res/15-35	2600 ± 117	21	0.7	0.5	0.0125	2728	2587
Res/18-35	2900 ± 122	34.8	0.7	0.7	0.0175	3217	2929
Res/55-75	3450 ± 149	60	0.88	0.7	0.0175	3604	3442
Res/0.5	2470 ± 104	15	0.4	0.8	0.02	2817	2480
Res/1.0	2380 ± 100	11	0.4	0.7	0.0175	2572	2382
Res/1.5	2500 ± 108	16	0.4	0.8	0.02	2817	2480

## Data Availability

The data sets analyzed during the current study are available from the corresponding author on reasonable request.
